# Magnetic Resonance Imaging Diagnosis of Metastatic Lymph Nodes in a Rabbit Model: Efficacy of PJY10, a New Ultrasmall Superparamagnetic Iron Oxide Agent, with Monodisperse Iron Oxide Core and Multiple-Interaction Ligands

**DOI:** 10.1371/journal.pone.0107583

**Published:** 2014-09-12

**Authors:** Roh-Eul Yoo, Seung Hong Choi, Hye Rim Cho, Bong-sik Jeon, Eunbyul Kwon, Eung-gyu Kim, Juyoung Park, Wan-Jae Myeong, Jae-Kyung Won, Yun-Sang Lee, Ji-Hoon Kim, Sun-Won Park, Chul-Ho Sohn

**Affiliations:** 1 Department of Radiology, Seoul National University College of Medicine, Seoul, Korea; 2 Center for Nanoparticle Research, Institute for Basic Science (IBS), Seoul National University, Seoul, Korea; 3 School of Chemical and Biological Engineering, Seoul National University, Seoul, Korea; 4 Department of Radiation Applied Life Science, Seoul National University College of Medicine, Seoul, Korea; 5 Research & Development Center, Hanwha Chemical Corp., Daejeon, Yuseong-gu, Korea; 6 Department of Pathology, Seoul National University College of Medicine, Seoul, Korea; 7 Department of Nuclear Medicine, Seoul National University College of Medicine, Seoul, Korea; 8 Department of Radiology, Boramae Medical Center, Seoul, Korea; NIH, United States of America

## Abstract

**Background:**

Accurate diagnosis of lymph node metastasis is crucial in treatment planning for cancer patients. Despite the use of various parameters, making correct diagnosis of a small metastatic or a hyperplastic benign node is still a challenge. In this study, we evaluated the feasibility of detecting lymph node metastasis using a new ultrasmall superparamagnetic iron oxide particle, PJY10, in a rabbit model.

**Methods:**

To make metastatic and benign lymph nodes, either VX2 carcinoma or fecal material suspension was inoculated into thighs of 56 rabbits three weeks or three days before magnetic resonance (MR) imaging, respectively. T2*-weighted 3T MR imaging was performed before and 24 hours after PJY10 injection (5.2 [n = 15], 7.8 [n = 17], and 10.4 [n = 24] mg Fe/kg). MR images were correlated with pathologic results to calculate sensitivity and specificity. Quantitative analysis of the signal intensity (SI) – number of voxels_[low]_ (the fraction of the number of voxels with the normalized SI on the postcontrast image lower than that on the precontrast image) and mean SI ratio – was also performed for each lymph node.

**Results:**

Sensitivities were 100% at all three dosages, whereas specificity increased with increasing dosage (89% at 10.4 mg Fe/kg). The benign nodes had a significantly higher number of voxels_[low]_ and a lower mean SI ratio than the metastatic nodes at the dosage of 10.4 mg Fe/kg (*P*<.001). Az values were 0.905 for the number of voxels_[low]_ and 0.952 for the mean SI ratio. The number of voxels_[low]_ (*P* = .019) and the mean SI ratio (*P* = .034) had significant correlations with the histopathologic area ratio of metastatic foci in the metastatic nodes at 10.4 mg Fe/kg.

**Conclusions:**

PJY10 enabled clear demonstration of lymph node metastasis with high sensitivity and specificity at its optimal dosage of 10.4 mg Fe/kg.

## Introduction

When a malignancy is diagnosed in a patient, decision on which path the patient is to take thereafter for the better treatment outcome heavily relies on the presence of the spread of primary malignancy to regional or distant lymph nodes. Albeit not as accurate as desired, cross-sectional imaging modalities such as computed tomography (CT) and magnetic resonance (MR) imaging have been the mainstay for assessing the local extent of primary malignancy as well as the regional lymph node involvement and distant metastases [1]. Traditionally, primary criterion for assessing the lymph node involvement has been the nodal size, with reported accuracies ranging from 63% to 75% for CT and 68% to 69% for MR imaging, respectively [1]. In an effort to improve the diagnostic accuracy, several studies have investigated various parameters – specifically, nodal morphology, border, signal intensity (SI) characteristics, enhancement kinetics, apparent diffusion coefficient, and 18F-fluorodeoxyglucose (18F-FDG) and 18F-choline uptakes – for their added values in diagnosing metastatic lymph nodes [2]–[10]. Although some promising results have been reported with the use of the parameters, the inherent limitation in diagnosing small metastatic or hyperplastic benign nodes has not been overcome, necessitating the development of a tissue-specific, highly sensitive and specific imaging technique to facilitate nodal characterization [1].

Ferumoxtran-10 (Combidex [Advanced Magnetics, Cambridge, MA, USA]; also known as Sinerem, AMI-7227, AMI-227, and BMS 180549) – one of the most popular ultrasmall superparamagnetic iron oxide (USPIO) particles – is a reticuloendothelial system-targeted MR contrast agent, specifically developed for MR lymphangiography [11]. Several studies on various body regions such as the head and neck, chest, abdomen, pelvis, and prostate demonstrated a significant improvement of the sensitivity and specificity in lymph node characterization after ferumoxtran-10 administration [12]. Furthermore, the contrast was reported to be clinically well-tolerated according to the comprehensive phase III efficacy and safety data, with the occurrence of only mild to moderate adverse events of short durations; therefore, it garnered substantial attention [13]. Nevertheless, ferumoxtran-10 failed to receive the final Food and Drug Administration (FDA) approval as the FDA’s Oncologic Drugs Advisory Committee concluded that clinical data at the time were insufficient to support a broad indication for use of ferumoxtran-10 across all cancer types.

In pursuit of a new USPIO contrast agent, we have synthesized PJY10 with multiple-interaction ligands inspired from a mussel adhesive protein [14]. For high T2 relaxivity, PJY10 was designed so that it could have a larger and more uniform iron oxide crystalline core than ferumoxtran-10 (13±2 nm, compared with 4.3–6.0 nm in ferumoxtran-10). Furthermore, unlike ferumoxtran-10, unique multiple-interaction ligands (MIL) capable of providing various binding modes were used in place of low-molecular-weight dextrans to enhance colloidal stability. The purpose of this study was to evaluate the feasibility of detecting lymph node metastasis using the new USPIO particle in a rabbit model.

## Materials and Methods

This experiment was approved by the Institutional Animal Care and Use Committee (IACUC) at Seoul National University Hospital.

### Animal Preparation

The experiments were performed using 56 New Zealand white rabbits weighing 2.5–3.0 kg. The animals were allowed food and water ad libitum.

To create an animal model of metastatic lymph nodes (ie, tumor model), the experimental VX2 carcinoma was prepared and 1 mL of tumor suspension was inoculated intramuscularly into either side of the rabbits’ thighs (n = 32), following the previously reported method [15]. To create an animal model of benign lymph nodes (ie, inflammation model), as a counterpart of the tumor model, we slightly modified the previously described method and used 1 mL of suspension prepared from the rabbits’ fecal material instead of egg-yolk emulsion for intramuscular inoculation [16].

For the tumor model, MR imaging with a 3.0T scanner was performed three weeks after the tumor inoculation. On the other hand, MR imaging was performed three days after the fecal material inoculation in the inflammation model.

### MR Contrast Material

PJY10, which is composed of a monodisperse iron oxide nanoparticle covered with unique multiple-interaction ligands (MILs) (ie, poly(ethylene glycol) (PEG) derivatives), was prepared according to the previously described method [14] with slight modifications and dispersed in a 0.9% sodium chloride solution. Structurally, MIL is a poly (L-3,4-dihydroxyphenylalanine) (polyDOPA) based ligand, consisting of methoxypoly (ethylene glycol) (mPEG) grafted cationic hyperbranched polyethylenimine (bPEI) and multi-initiated peptide domain of the polyDOPA [14]. With respect to the particle size dispersity, the core size of the iron oxide nanoparticle was determined to be 13.0±1.2 nm by a 200 kV Field-emission Transmission Electron Microscope (FE-TEM) (JEM-2100F [HR]; JEOL Ltd., Tokyo, Japan) (see also Figure S1). The particles were highly dispersible in aqueous conditions, and the hydrodynamic diameter was found to be 25–30 nm by means of dynamic light scattering (DLS, Nano ZS; Malvern Instrument Ltd., Malvern, England). The hydrodynamic diameter of PJY10 satisfied the recommended size criteria for lymphotrophic superparamagnetic nanoparticle (ie, less than 30 nm) [17]. Furthermore, the optimal core size was determined from our preliminary study using nude mice. The T2 relaxivities of PJY10 measured using 1.5T (Signa HDXt or Signa Excite; GE Medical Systems, Milwaukee, Wis), 3.0T (TrioTim; Siemens, Erlangen, Germany), and 4.7T (Biospec 47/40; Bruker Medical Systems, Ettlingen, Germany) MR scanners were 123, 167, and 281 mM^−1^s^−1^, respectively. The concentration of iron in PJY10 was 20 mg Fe/mL. We also evaluated pharmacokinetic properties of PJY10 using rats and mice (see Text S1, Table S1, and Figures S2 and S3).

### MR Imaging Scanning

During MR image acquisition, the animals were sedated with an intramuscular injection of 50 mg of ketamine hydrochloride and 20 mg of xylazine hydrochloride per kilogram of body weight. In all 56 rabbits, MR imaging was performed with a 3.0T MR scanner (TrioTim; Siemens, Erlangen, Germany) and a knee coil to improve the resolution. In addition, the sigmoid colon and the rectum were filled with 80 mL of normal saline to minimize susceptibility artifacts due to the bowel gas.

At day one, after routine localization images, precontrast coronal T2*-weighted three-dimensional (3D) gradient-echo sequences were acquired. Based on the results from our preliminary study using mice, where the PJY10 concentration of 10.4 mg Fe/kg was shown to be the saturation dose (ie, the dose at which the signal reduction of lymph nodes between pre- and postcontrast images reached the maximum), PJY10 was prepared in three different concentrations, 5.2, 7.8, and 10.4 mg Fe/kg. Subsequently, PJY10 was intravenously administered via the ear veins of the rabbits. Postcontrast coronal T2*-weighted 3D gradient-echo sequences were acquired 24 hours after the contrast administration. The scans covered the entire region from the mid-abdomen to the upper thigh. The animals were imaged in the prone position. Imaging parameters including repetition time, echo time, number of signals acquired, and section thickness were modified to find the optimized values for accurate depiction of pelvic lymph nodes. Repetition time was varied from 51 to 400 msec, followed by echo time, which was varied from 15 to 24 msec. Section thickness was varied from 1 to 2 mm. Number of signals acquired was varied from two to four. Finally, two board-certified radiologists (S.H.C. and J.H.K. with seven and 15 years of experience in the interpretation of MR imaging studies) reviewed the images obtained using the various parameters and the optimal parameters were chosen as follows: repetition time (msec)/echo time (msec), 51/22; flip angle, 12°; echo train length, 1; section thickness, 1 mm; field of view (FOV), 120×84 mm; matrix, 256×180; and number of signals acquired, 4.

### Isolation of Lymph Nodes and Histopathologic Examination

After the acquisition of the postcontrast T2*-weighted MR images, the rabbits were sacrificed with a lethal dose (90 mg/kg) of intravenously administered sodium pentobarbital. The iliac and retroperitoneal lymph nodes were isolated by one author (R.E.Y.). The lymph nodes were dissected with the surrounding soft tissue at the iliac bifurcation in an en bloc fashion. Based on the MR image review prior to the lymph node dissection, the lymph nodes were labeled accordingly for the MR imaging-histopathologic correlation and fixed with 10% formalin. The lymph nodes were embedded in paraffin and then sectioned at 0.5-mm intervals. From each 0.5-mm section, a 5-µm thick slide was prepared in the coronal plane for hematoxylin-eosin (H-E) staining and subsequently examined by a pathologist (J.K.W.) for the presence of metastasis.

For quantitative analysis, the long- and short-axis diameters of each lymph node were measured in the largest section, while the maximum diameters of metastatic foci were measured on representative slides. In some lymph nodes, the largest metastatic focus was not found in the largest section. For each metastatic lymph node, the following formula was used to calculate the area ratio between the metastatic foci and the overall lymph node tissue:

Area_ratio_ = ∑(long diameter×short diameter)_metastatic foci_/(long diameter×short diameter)_overall lymph node tissue_ [18].

### Image Analysis

#### Qualitative Analysis for Differentiation of the Benign and Malignant Lymph Nodes

The qualitative analysis was performed by two investigators (S.H.C. and J.H.K. with seven and 15 years of experience in the interpretation of MR imaging studies), who were blinded to the histopathologic information. The MR images were presented in random order by one author (R.E.Y.) and reviewed on a picture archiving and communication system workstation. A diagnosis of malignancy on the postcontrast T2*-weighted MR images was made by consensus, using the following criteria reported in previous studies [13], [19], [20]: a lymph node with an overall high SI, an eccentric or central high SI with darkening along the peripheral rim was considered to be malignant. On the other hand, a node with an overall dark SI, an overall dark SI apart from the fatty hila, an overall dark SI with tiny speckles of high SI, or a central low SI was considered to be nonmalignant.

#### Quantitative Analysis of the SI in Each Lymph Node

The SI of each lymph node was quantitatively analyzed by one author (R.E.Y.). Regions of interest (ROIs) that contained the entire lymph node were drawn in each section of the postcontrast T2*-weighted MR images, using Image-J software (National Institutes of Health, Bethesda, Md). The data acquired from each section were summated to derive voxel-by-voxel SIs for the entire lymph node by using software developed in-house. At the same time, the overall mean SI of each lymph node on the precontrast MR image was measured by drawing the ROI along the margin of the lymph node on its representative section.

Given the earlier findings that the muscle tissue remains unchanged by the contrast agent [21], [22], SI of the left thigh muscle was measured at a single ROI (5–7 mm^2^ in size) on both the pre- and postcontrast MR images. Subsequently, the SI values of each voxel on the postcontrast MR image and the overall mean SI of the lymph node on the precontrast image were divided by the muscle SI to cancel the SI fluctuations related to variations in technical parameters between the pre- and postcontrast images.

Finally, the fraction of the number of voxels with the normalized SI on the postcontrast image lower than that on the precontrast image (ie, number of voxels_[low]_) was determined. Additionally, the mean SI ratio between the pre- and postcontrast images was calculated using the following formula: SI ratio = (SI lymph node_[post-USPIO]_/SI muscle_[post-USPIO]_)/(SI lymph node_[pre-USPIO]_/SI muscle_[pre-USPIO]_).

### Statistical Analysis

Statistical analyses were performed by using commercially available software (MedCalc, version 8.0.0.1; MedCalc software, Mariakerke, Belgium). The data for each parameter was assessed for normality with the Kolmogorov-Smirnov test. In all tests, *P* values less than.05 were considered statistically significant.

#### Qualitative Analysis for Differentiation of the Benign and Metastatic Lymph Nodes

Sensitivity and specificity for differentiation of the benign and metastatic lymph nodes were calculated for each PJY10 dosage (5.2, 7.8, 10.4 mg Fe/kg). Data clustering (ie, more than one lesion per rabbit) was accounted for with the method of Rao and Scott [23].

#### Quantitative Analysis of the SI in Each Lymph Node

For each contrast dosage, the unpaired Student t test was used to assess whether the number of voxels_[low]_ and the mean SI ratio of the benign lymph nodes differed significantly from those of the metastatic lymph nodes.

The receiver operating characteristic (ROC) curve analysis was performed to estimate the area under the curve (Az) based on the number of voxels_[low]_ and the mean SI ratio.

As for the metastatic lymph nodes, Pearson correlation analysis was performed to measure the significance of the association between the histopathologic area ratio (Area_ratio_) and either the number of voxels_[low]_ or the mean SI ratio for each contrast dosage. Regression analysis of the data was performed using the general linear model.

#### Diagnostic Yields at the Optimal (Lowest Effective) Dosage

Regarding the optimal (lowest effective) contrast dosage, ROC curves were constructed for both the number of voxels_[low]_ and the mean SI ratio to determine cutoff levels that provided a balance between sensitivity and specificity for the diagnosis of lymph node metastasis. The sensitivities and specificities at the optimal cutoff levels as well as the Az values were calculated, along with 95% confidence intervals (CIs).

## Results

### Histopathology

The numbers of benign and metastatic lymph nodes isolated from the rabbit models (ie, tumor and inflammation models) are summarized for each PJY10 dosage in Table 1. 10.4 mg Fe/kg of PJY10 was administered to 24 rabbits (12 tumor models and 12 inflammation models), whereas 7.8 mg Fe/kg of PJY10 was administered to 17 rabbits (11 tumor models and 6 inflammation models). 5.2 mg Fe/kg of PJY10 was administered to 15 rabbits (9 tumor models and 6 inflammation models). The mean long- and short-axis diameters of the benign lymph nodes were 8.0±3.2 and 3.6±1.4 mm. In comparison, the mean long- and short-axis diameters of the metastatic lymph nodes were 10.6±3.8 and 6.6±2.4 mm. Moreover, the mean long- and short-axis diameters of the metastatic foci were 2.5±2.8 and 1.7±1.9 mm.

**Table 1:** Number of all removed lymph nodes identified on histopathologic specimens.

	PJY10 dosage (mg Fe/kg)		
	5.2	7.8	10. 4
Number of rabbits	15	17	24
Number of benign nodes	45	51	53
Number of metastatic nodes	20	17	18

### MR Image Analysis

#### Qualitative Analysis for Differentiation of the Benign and Malignant Lymph Nodes

Sensitivity and specificity at each PJY10 dosage are provided in Table 2. The sensitivity was 100% (20 of 20, 17 of 17, and 18 of 18 in 5.2, 7.8, and 10.4 mg Fe/kg, respectively) in all three dosages, whereas the specificity increased with increasing dosage, with the dosage of 10.4 mg Fe/kg having the highest specificity of 89% (47 of 53).

**Table 2:** Sensitivity and specificity at each PJY10 dosage.

	PJY10 dosage (mg Fe/kg)		
	5.2	7.8	10.4
Sensitivity (%) [95% CI]	100 (20 of 20) [74, 100]	100 (17 of 17) [77, 100]	100 (18 of 18) [74, 100]
Specificity (%) [95% CI]	62 (28 of 45) [45, 79]	71 (36 of 51) [58, 83]	89 (47 of 53) [78, 99]

Data clustering (ie, more than one lesion per rabbit) was accounted for with the method of Rao and Scott [22]. Data in parentheses are the raw data used to calculate the percentages, and data in square brackets are 95% confidence intervals (CI) expressed as percentages.

#### Quantitative Analysis of the SI in Each Lymph Node

The mean SI ratio in the benign lymph nodes was significantly lower than that in the malignant lymph nodes at the dosages of 7.8 and 10.4 mg Fe/kg (*P*<.001 for both). Accordingly, the number of voxels_[low]_ was significantly higher for the benign lymph nodes than for the metastatic lymph nodes at the dosage of 10.4 mg Fe/kg (*P*<.001). The benign lymph nodes had a higher number of voxels_[low]_ than the metastatic lymph nodes at the dosage of 7.8 mg Fe/kg, although the level did not achieve statistical significance (*P* = .077) (Table 3).

**Table 3:** Quantitative analysis: signal intensity difference between the benign and metastatic lymph nodes at different PJY10 dosages.

	PJY10 dosage (mg Fe/kg)		
	5.2	7.8	10.4
Number of voxels_[low]_ *			
Benign nodes	0.916 (n = 45)	0.931 (n = 51)	0.989 (n = 53)
Metastatic nodes	0.869 (n = 20)	0.823 (n = 17)	0.831 (n = 18)
*P* value	.240	.077	<.001
Mean SI ratio†			
Benign nodes	0.619 (n = 45)	0.560 (n = 51)	0.407 (n = 53)
Metastatic nodes	0.716 (n = 20)	0.777 (n = 17)	0.749 (n = 18)
*P* value	.070	<.001	<.001

Data in parentheses are the number of lymph nodes.

*The number of voxels_[low]_ represents the fraction of the number of voxels with the normalized SI on the postcontrast image lower than that on the precontrast image.

† The mean signal intensity (SI) ratio between pre- and postcontrast images is calculated using the following formula: SI ratio = (SI lymph node_[post-USPIO]_/SI muscle_[post-USPIO]_)/(SI lymph node_[pre-USPIO]_/SI muscle_[pre-USPIO]_).

With regard to diagnosing lymph node metastasis, the Az values from the ROC analysis tended to increase for both the number of voxels_[low]_ and the mean SI ratio, with increasing PJY10 dosage (Table 4).

**Table 4:** Quantitative analysis: Az values with regard to diagnosing lymph node metastasis at different PJY10 dosages.

	PJY10 dosage (mg Fe/kg)		
	5.2	7.8	10.4
Number of voxels_[low]_ [95% CI]*	0.647 [0.518, 0.761]	0.779 [0.661, 0.870]	0.905 [0.812, 0.962]
Mean SI ratio [95% CI]†	0.627 [0.498, 0.744]	0.785 [0.669, 0.876]	0.952 [0.873, 0.988]

Data in square brackets are 95% confidence intervals (CI) expressed as percentages.

*The number of voxels_[low]_ represents the fraction of the number of voxels with the normalized SI on the postcontrast image lower than that on the precontrast image.

† The mean signal intensity (SI) ratio between pre- and postcontrast images is calculated using the following formula: SI ratio = (SI lymph node_[post-USPIO]_/SI muscle_[post-USPIO]_)/(SI lymph node_[pre-USPIO]_/SI muscle_[pre-USPIO]_).

**Figure 1:**
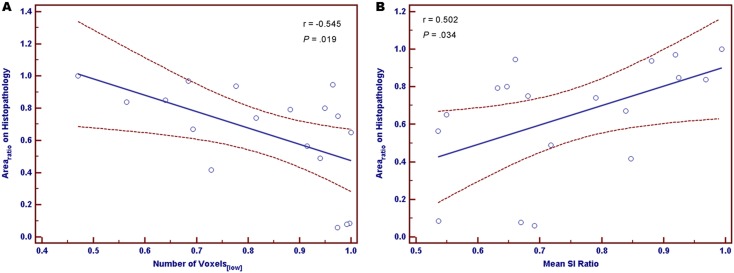
Linear regression plots between histopathologic area ratio (Area_ratio_) and quantitative image analysis parameters. The best-fit lines (ie, the graphs of the linear regression equations) are shown as solid lines for linear regression analyses between the Area_ratio_ and either the number of voxels_[low]_ (A) or the mean signal intensity (SI) ratio (B) at PJY10 dosage of 10.4 mg Fe/kg. The curves above and below the best-fit line represent the upper and lower bounds of the 95% confidence interval (CI). r = correlation coefficient.

Both the number of voxels_[low]_ and the mean SI ratio had statistically significant correlations with the histopathologic area ratio (Area_ratio_) only at the dosage of 10.4 mg Fe/kg (r = −0.142, *P* = .552; r = 0.233, *P* = .322 for 5.2 mg Fe/kg; r = −0.034, *P* = .897; r = 0.114, *P* = .664 for 7.8 mg Fe/kg; r = −0.545, *P* = .019; r = 0.502, *P* = .034 for 10.4 mg Fe/kg) (Figure 1).

#### Diagnostic Yields at the Optimal (Lowest Effective) Dosage of 10

**Figure 2:**
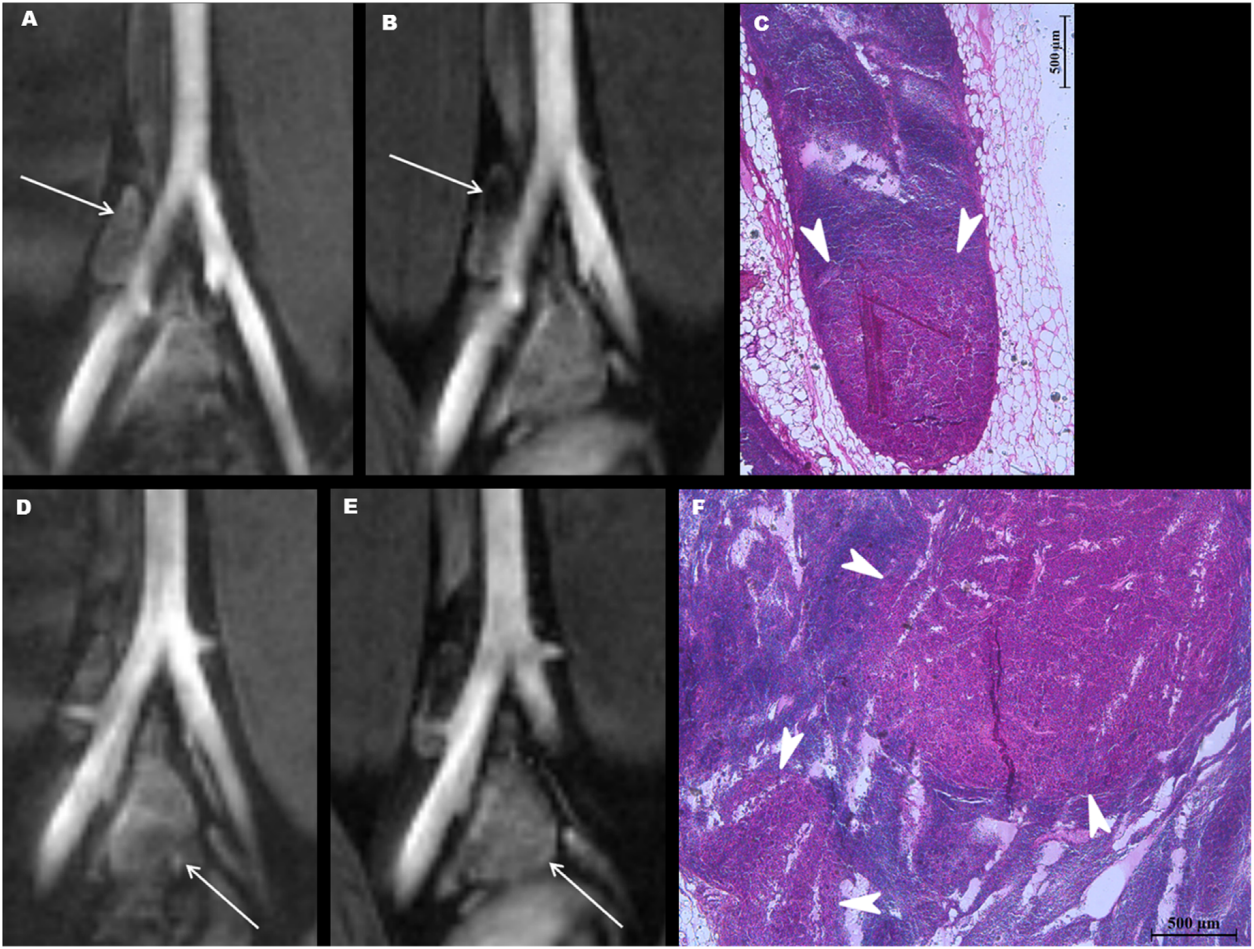
A tumor model into which 10.4 mg Fe/kg of PJY10 was administered. (A, B) An ovoid right common iliac lymph node with a short-axis diameter of 0.3 cm shows a focal signal drop at its superior aspect (arrow) on the postcontrast coronal T2*-weighted MR image (B), as compared with the precontrast image (A). (C) The hematoxylin-eosin (H-E) stained pathology specimen at high-power field (magnification×400) well-demonstrates a metastatic focus (arrowheads) at the inferior aspect of the lymph node, corresponding to the portion with no signal drop on the postcontrast MR image (B). (D, E) Another enlarged lymph node (arrow) at the iliac bifurcation remains unchanged on the postcontrast coronal T2*-weighted MR image (E), as compared with the precontrast image (D). (Some susceptibility artifact due to adjacent bowel gas is present on the precontrast image.) (F) Multiple metastatic foci (arrowheads) were confirmed on the hematoxylin-eosin (H-E) stained pathology specimen (magnification×400).

**Figure 3:**
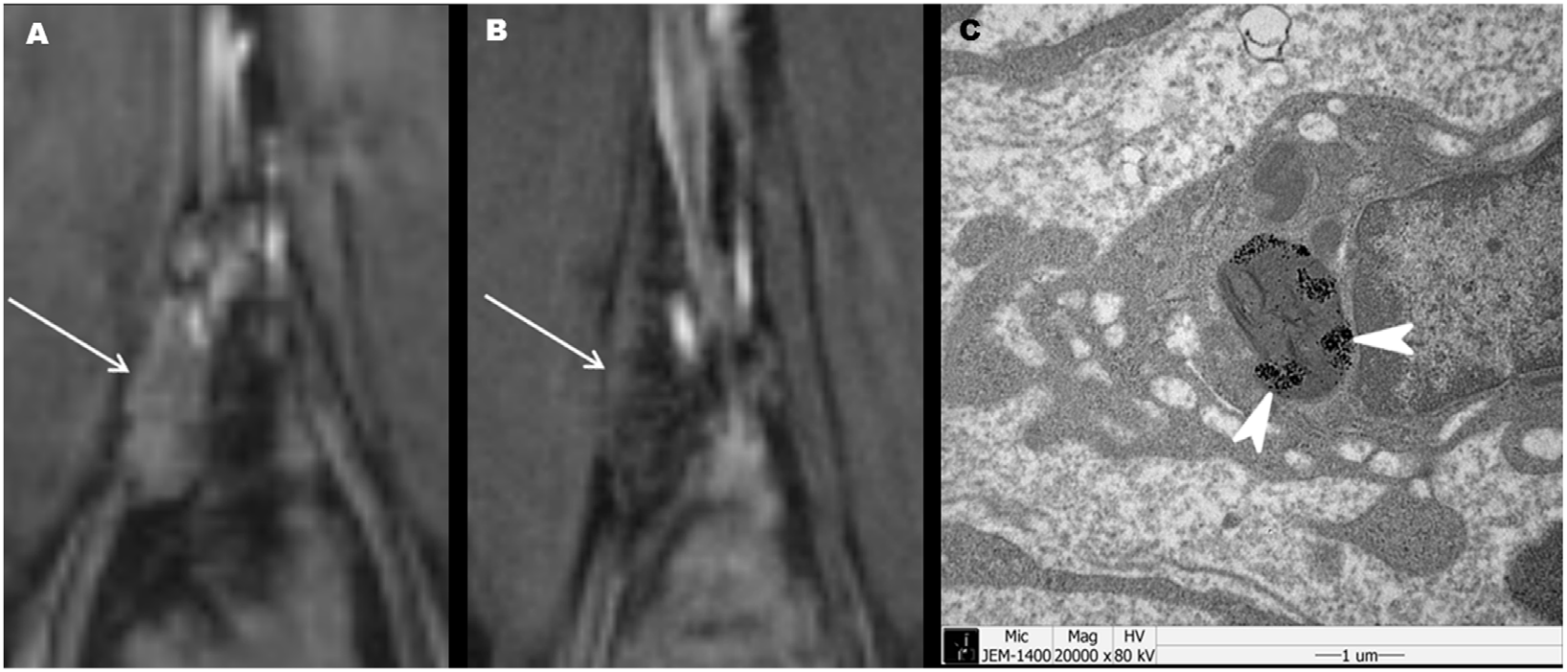
An inflammation model into which 10.4 mg Fe/kg of PJY10 was administered. (A, B) The pre- (A) and postcontrast (B) coronal T2*-weighted MR images demonstrate a central darkening of an enlarged lymph node (arrow) in the right common iliac area. (C) An electron microscopy image of the lymph node confirmed the presence of contrast particles (arrowheads) within the cytoplasmic organelle of the lymph node macrophage (magnification×20,000).

**4** **mg Fe/kg.** According to the results presented in Figure 1 and Tables 2–4, the concentration of 10.4 mg Fe/kg was selected as the optimal (lowest effective) dosage of PJY10 for diagnosing lymph node metastasis. Representative cases of the metastatic and benign lymph nodes at the dosage of 10.4 mg Fe/kg are shown in Figures 2 and 3.

At the dosage of 10.4 mg Fe/kg, the cutoff values that provided a balance between sensitivity and specificity for the diagnosis of lymph node metastasis were 0.992 for the number of voxels_[low]_ and 0.533 for the mean SI ratio. The corresponding sensitivities and specificities at the cutoff values were 89% (95% CI: 65, 99) and 81% (95% CI: 68, 91) for the number of voxels_[low]_ and 100% (95% CI: 82, 100) and 81% (95% CI: 68, 91) for the mean SI ratio. The Az values were 0.905 (95% CI: 0.812, 0.962) and 0.952 (95% CI: 0.873, 0.988) for the number of voxels_[low]_ and the mean SI ratio, respectively.

## Discussion

The present study evaluated the feasibility of detecting lymph node metastasis in a rabbit model using PJY10, a new USPIO contrast agent with a monodisperse iron oxide core and multiple-interaction ligands. The use of the contrast dosage of 10.4 mg Fe/kg yielded the sensitivity of 100% and specificity of 89% in the subjective analysis based on the previously reported criteria [13], [19], [20]. Moreover, the number of voxels_[low]_ and the mean SI ratio were used to measure the SI difference between the benign and metastatic lymph nodes, and the benign lymph nodes were shown to have a significantly higher number of voxels_[low]_ and a lower mean SI ratio compared with the metastatic lymph nodes. At the optimal (lowest effective) dosage of 10.4 mg Fe/kg, the Az values were 0.905 for the number of voxels_[low]_ and 0.952 for the mean SI ratio. The number of voxels_[low]_ and the mean SI ratio also had significant positive correlations with the histopathologic area ratio between the metastatic foci and the overall lymph node tissue.

Ever since the introduction of contrast-enhanced MR lymphography, a number of diverse compounds have been rigorously developed for varying routes of administration, ie, direct or endolymphatic, indirect or interstitial, and intravenous (IV) injection [24]. Compared with the interstitial route of administration, the IV injection is advantageous in that it yields a higher reproducibility in the imaging performance and allows evaluation of distant as well as regional lymph nodes with a single injection [24].

USPIO particles were the first contrast media used for the IV MR lymphography. After IV administration, these compounds are transported to the lymph nodes by the lymphatic vessels and phagocytosed by functional macrophages in the nonmetastatic lymph node tissue [24], [25]. Once accumulated in the functional macrophages, the T2* effect of the iron oxide particles causes the signal drop of the nonmetastatic lymph nodes on the USPIO-enhanced MR image. In contrast, the SI of the metastatic lymph nodes deprived of the functional macrophages remains unchanged [24]. Thus, USPIO-enhanced MR imaging is believed to enable identification of metastatic areas within the lymph nodes independently of the lymph node size [26], [27].

Two fundamental prerequisites for the successful biomedical use of the USPIO particles have been reported: 1) strong T2 relaxivity, which leads to a marked decrease in SI (negative enhancement) on the postcontrast image and 2) colloidal stability in harsh biological environments [14], [28]. Ferumoxtran-10 (Combidex; Advanced Magnetics, Cambridge, MA, USA), most widely evaluated first-generation USPIO particle, is 20–50 nm (mean 35 nm) in size, and composed of an iron oxide crystalline core of 4.3–6.0 nm covered by low-molecular-weight dextran. The T1 and T2 relaxivities of the compound were found to be 23 mM^−1^s^−1^ and 53 mM^−1^s^−1^ (20 MHz, 39°C), respectively, in 0.5% agar [29]. A meta-analysis of the diagnostic precision of ferumoxtran-10-enhanced MR imaging for lymph node metastasis has shown an overall sensitivity of 0.90 and specificity of 0.96 [12].

The pursuit of a new USPIO contrast agent with strong T2 relaxivity and high colloidal stability led to the development of the new USPIO contrast agent, PJY10. First, PJY10 was found to have a T2 relaxivity of 281 mM^−1^s^−1^ at 4.7T, which was three times higher than that of Combidex (94 mM^−1^s^−1^) under the same conditions. T2 relaxivities of PJY10 measured at 1.5 and 3.0T (ie, 123 and 167 mM^−1^s^−1^, respectively) were also higher than those of Combidex reported in the previous literature (ie, 100 and 128 mM^−1^s^−1^, respectively) [28]. The higher T2 relaxivity of PJY10 can be attributed to its larger and more uniform core (13±2 nm, compared with 4.3–6.0 nm in Combidex), along with its higher crystallinity, as T2 relaxivity (R_2_) of the nanoparticles is defined by
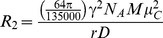
where N_A_ is Avogadro’s number, M is the particle molarity (moles L^−1^), µ_c_ is the magnetic moment of the nanoparticle, which is proportional to its volume, r is the effective radius of the nanoparticle, and D is the diffusion coefficient of water molecules. In order to obtain the large, uniform core and high crystallinity, thermal decomposition method was employed instead of coprecipitation method which has been used for the conventional T2 agents [30]–[32]. Overall size of PJY10, however, was designed to be small enough as 25–30 nm to ensure a longer blood half-life, based on the fact that the particle size is the main factor that has a major influence on its blood half-life and biodistribution. Small nanoparticles are known to have a longer plasma-circulation time due to their slow excretion by the liver, thus suggesting the recommended size criteria for lymphotrophic superparamagnetic nanoparticles to be less than 30 nm [33].

Second, a unique multiple-interaction ligand (MIL), inspired from a mussel adhesive protein, was developed to enhance colloidal stability [14]. Each component of the MIL contributes to the overall colloidal stability by providing various binding modes. First, the polyDOPA domain and the primary amine end groups of the MIL enable the simultaneous binding of multiple catechols and amines onto the surface of the hydrophobic nanoparticles [34]. Second, the amphiphilic hyperbranched block copolymer structure with both hydrophobic polyDOPA and hydrophilic PEG groups generates micelles encapsulating the nanoparticles. Third, the positively charged bPEI moiety can interact electrostatically with negatively charged metals and metal oxides of the nanoparticles [14]. Enhanced stability via the various binding modes was reflected in the results of our in vitro stability test, which proved that the new USPIO particle was highly stable at various salt concentrations, pH levels, temperatures, and in a high iron concentration of 15 mg Fe/mL [14].

Our new USPIO contrast agent with the specific design for the optimal T2 relaxivity and colloidal stability showed a high sensitivity and specificity (100% and 89%, respectively) for diagnosing lymph node metastasis at the dosage of 10.4 mg Fe/kg. Our preliminary study comparing PJY10 with MION-47– experimental nanoparticle highly similar to ferumoxtran-10 [35]–[37] – has shown that sensitivity of PJY10 at 10.4 mg Fe/kg was comparable to that of MION-47 at 2.6 mg Fe/kg (*P* = .064) (see Text S1 and Figure S4).

Although the most commonly used diagnostic guideline for the interpretation of USPIO-enhanced MR images is based on the patterns of contrast enhancement, such practice has a major drawback in that it inevitably involves a certain level of subjectivity, as noted by Islam and Harisinghani [1]. Instead, Lahaye et al [38] used the estimation of percentage of high SI within the node as a measure of lymph node metastasis in primary rectal cancer and found a cut-off value of 30% to be highly predictive for an involved node, with a sensitivity of 93% and a specificity of 96%. Similarly, a previous study using MION-47 has shown that the mean SI ratio between pre- and postcontrast images was a useful parameter in the differential diagnosis of the benign and metastatic lymph nodes, with a cut-off value of 0.533 resulting in a sensitivity of 93% and a specificity of 83% [18]. In the present study, we evaluated the mean SI ratio as a complement to our qualitative analysis based on the previously reported diagnostic guideline. The metastatic lymph nodes were shown to have a significantly higher mean SI ratio than the benign lymph nodes (*P*<.001), with a cut-off value of 0.533 at the contrast dosage of 10.4 mg Fe/kg yielding a sensitivity of 100% and a specificity of 81%. Furthermore, our study demonstrated that the mean SI ratio had a significant positive correlation with the histopathologic area ratio between the metastatic foci and the overall lymph node tissue in the metastatic lymph nodes, implying that the mean SI ratio may be a useful imaging biomarker for the tumor burden.

Our study had a few limitations. First, T1- and T2-weighted images, known to enhance the visualization of anatomy including the fatty hila, were not performed in this study [1]. Nonetheless, pre- and postcontrast T2*-weighted gradient-echo sequences have been shown to be the key sequences for displaying USPIO particle uptakes in lymph nodes and the imaging parameters in our study were optimized in a way that the acquisition of the T2*-weighted 3D gradient-echo sequences alone was adequate for delineation of the relevant anatomy. Second, the need for two MR imaging scans 24 hours apart could be a limitation in terms of time and costs, as in the case of ferumoxtran-10. However, Harisinghani et al [39] found no significant difference in the diagnostic precision between paired MR imaging (unenhanced MR imaging followed by ferumoxtran-10-enhanced MR imaging) and postcontrast MR imaging alone for an experienced reviewer. Further study is warranted to assess whether the PJY10-enhanced MR imaging alone may as well be sufficient to diagnose lymph node metastasis. Third, Prussian blue staining for direct visualization of iron oxide particles was not available in some lymph nodes, because of the limitation in number of sections that could be produced from relatively small pathology specimens (see Figure S5 for Prussian blue staining of a benign lymph node in an inflammation model). Fourth, the PJY10 dose used in this study was relatively higher than the clinical dose of ferumoxtran-10 (ie, 2.6 mg Fe/kg). The result may imply that the fraction of iron oxide particles, which get accumulated in lymph nodes, is lower in PJY10 compared with ferumoxtran-10 for the equal amount injected. This could be an intrinsic limitation of PJY10; nonetheless, no definite evidence of toxicity was found at the dose of 10.4 mg Fe/kg. Fifth, the saturation dose used as the basis of dose adjustment in this study was adopted from our preliminary study using nude mice. The saturation dose may be influenced by physiology of specific animals. That said, the same saturation dose was used because it is difficult to produce the experimental USPIO nanoparticle in a large-scale in a laboratory.

In conclusion, our experimental study with a rabbit model has demonstrated that PJY10, the new USPIO contrast agent with high T2 relaxivity and colloidal stability, may be a promising agent in the oncologic field by enabling clear demonstration of lymph node metastasis with high sensitivity and specificity at its optimal (lowest effective) dosage of 10.4 mg Fe/kg.

## Supporting Information

Figure S1 **An electron microscopy image of PJY10 nanoparticle (magnification×100,000).**
(TIF)

Figure S2 **Plasma concentration-time curves of PJY10 in Sprague-Dawley (SD) male rats weighing 260–340** **g.**
(TIF)

Figure S3 **Ratio of the residual ^59^Fe to the administered ^59^Fe (ie, %ID) for a specific organ with respect to the time after PJY10 administration.** (A) %ID per organ as a whole (%ID/organ) according to the time. (B) %ID per gram of the organ (%ID/g) according to the time.
(TIF)

Figure S4 **A tumor model (New Zealand white rabbit weighing 2.5–3.0 kg) into which 2.6 mg Fe/kg of MION-47 was administered.** (A, B) An enlarged lymph node at the iliac bifurcation (arrows) shows no discernible signal drop on the postcontrast coronal T2*-weighted MR image (B), as compared with the precontrast image (A). (C) The hematoxylin-eosin (H-E) stained pathology specimen revealed multiple large metastatic foci (arrowheads) (magnification×400).
(TIF)

Figure S5 **Prussian blue staining of a benign lymph node in an inflammation model, into which 10.4 mg Fe/kg of PJY10 was administered.** Iron oxide particles within functional macrophages are stained blue (magnification×400).
(TIF)

Table S1 Pharmacokinetic Parameters of PJY10.
(DOCX)

Text S1 **Pharmacokinetic Properties of PJY10.** Sensitivity and Specificity for Detection of Lymph Node Metastasis: Comparison of PJY10 with MION-47.
(DOCX)
