# Supplementary figures and images for: Magnetic Resonance Imaging Diagnosis of Metastatic Lymph Nodes in a Rabbit Model: Efficacy of PJY10, a New Ultrasmall Superparamagnetic Iron Oxide Agent, with Monodisperse Iron Oxide Core and Multiple-Interaction Ligands
Source: PLoS One. 2014 Sep 12;9(9):e107583. doi: 10.1371/journal.pone.0107583 (PMC4162649; doi:10.1371/journal.pone.0107583)

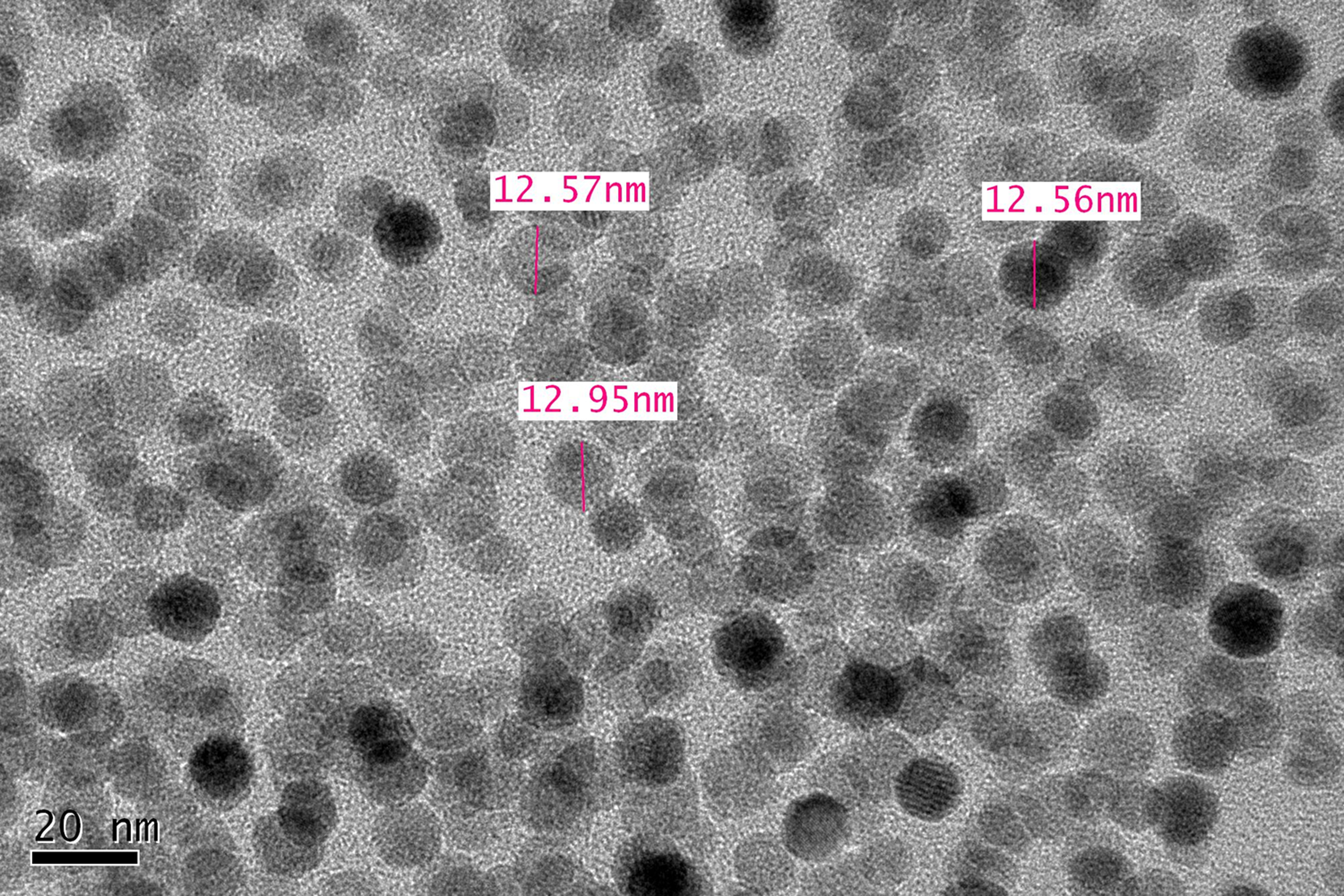

Supplement: Figure S1 — An electron microscopy image of PJY10 nanoparticle (magnification×100,000). (TIF) [file pone.0107583.s001.tif]

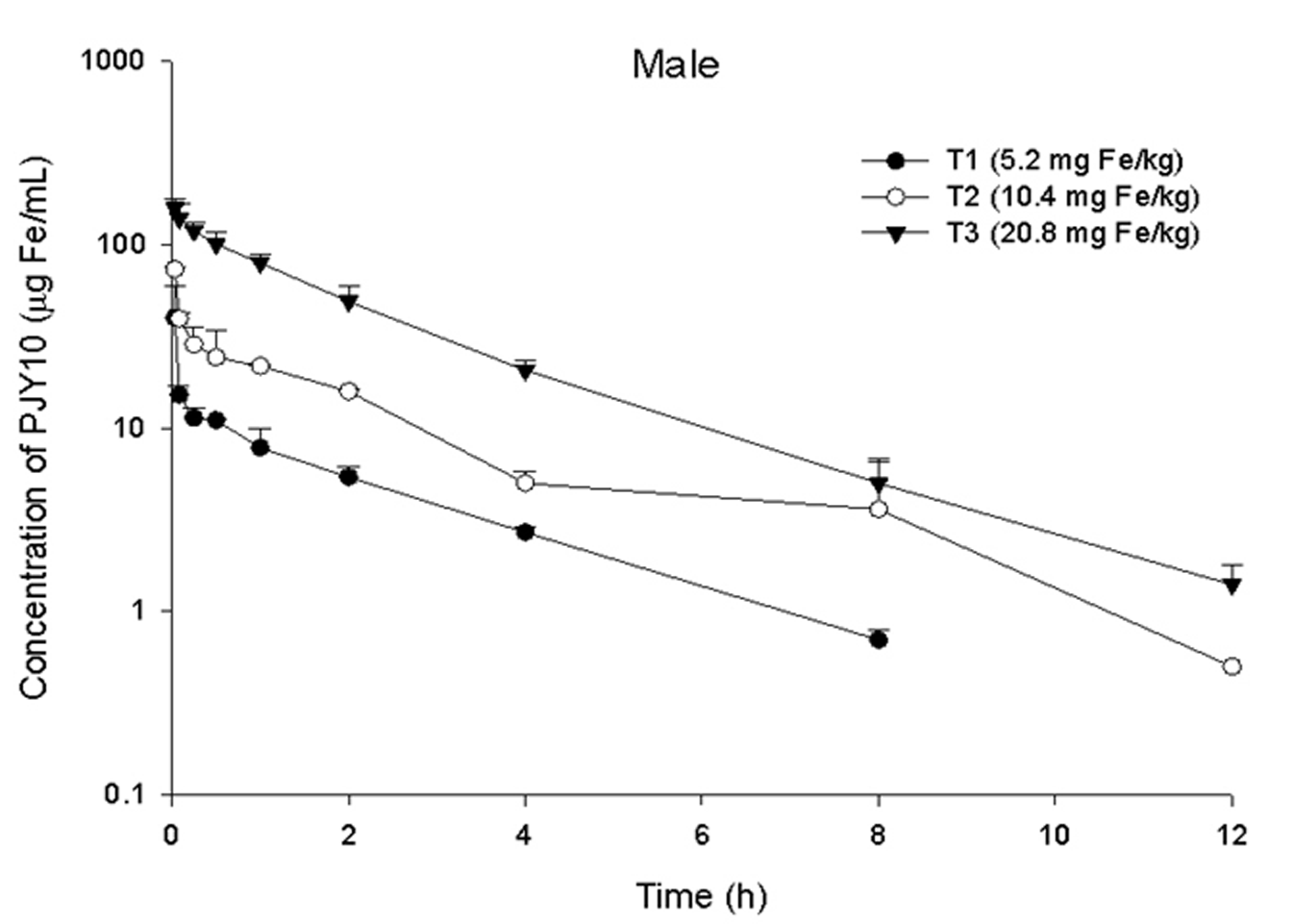

Supplement: Figure S2 — Plasma concentration-time curves of PJY10 in Sprague-Dawley (SD) male rats weighing 260–340 g. (TIF) [file pone.0107583.s002.tif]

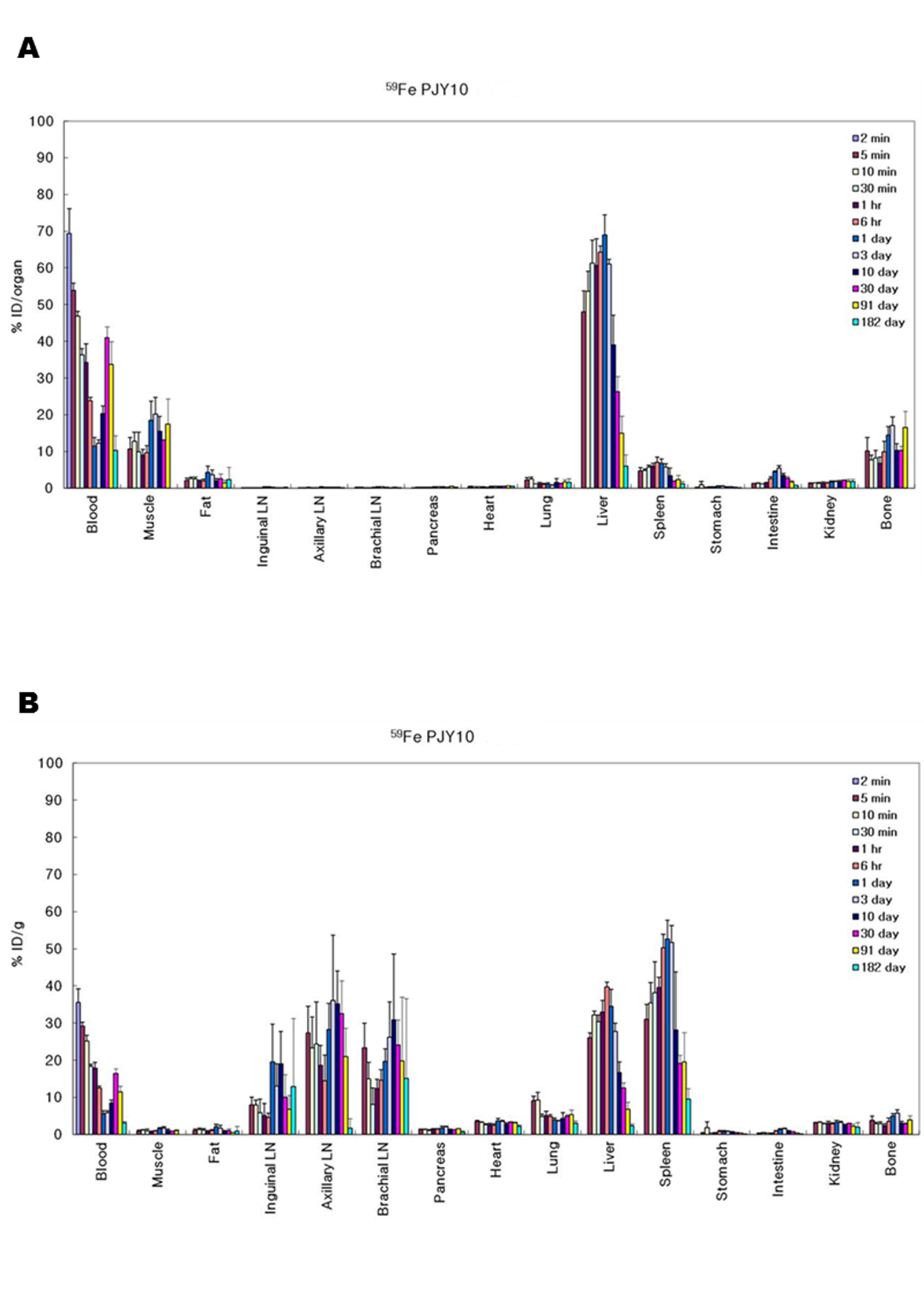

Supplement: Figure S3 — Ratio of the residual 59Fe to the administered 59Fe (ie, %ID) for a specific organ with respect to the time after PJY10 administration. (A) %ID per organ as a whole (%ID/organ) according to the time. (B) %ID per gram of the organ (%ID/g) according to the time. (TIF) [file pone.0107583.s003.tif]

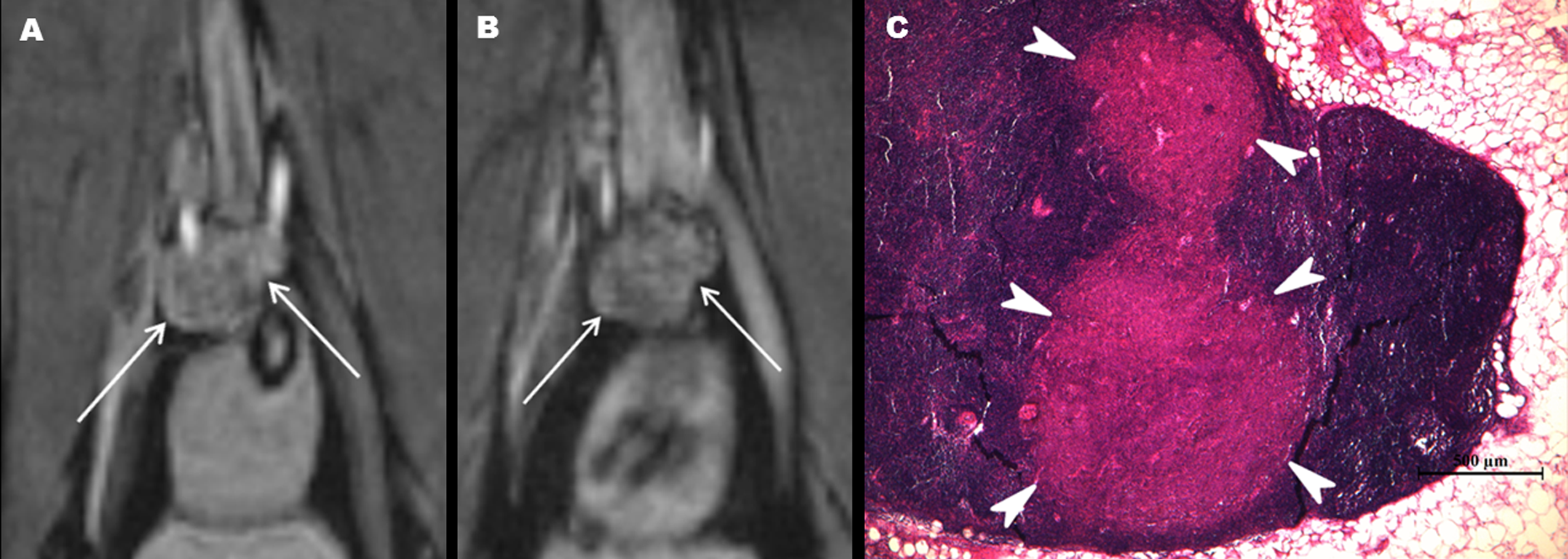

Supplement: Figure S4 — A tumor model (New Zealand white rabbit weighing 2.5–3.0 kg) into which 2.6 mg Fe/kg of MION-47 was administered. (A, B) An enlarged lymph node at the iliac bifurcation (arrows) shows no discernible signal drop on the postcontrast coronal T2*-weighted MR image (B), as compared with the precontrast image (A). (C) The hematoxylin-eosin (H-E) stained pathology specimen revealed multiple large metastatic foci (arrowheads) (magnification×400). (TIF) [file pone.0107583.s004.tif]

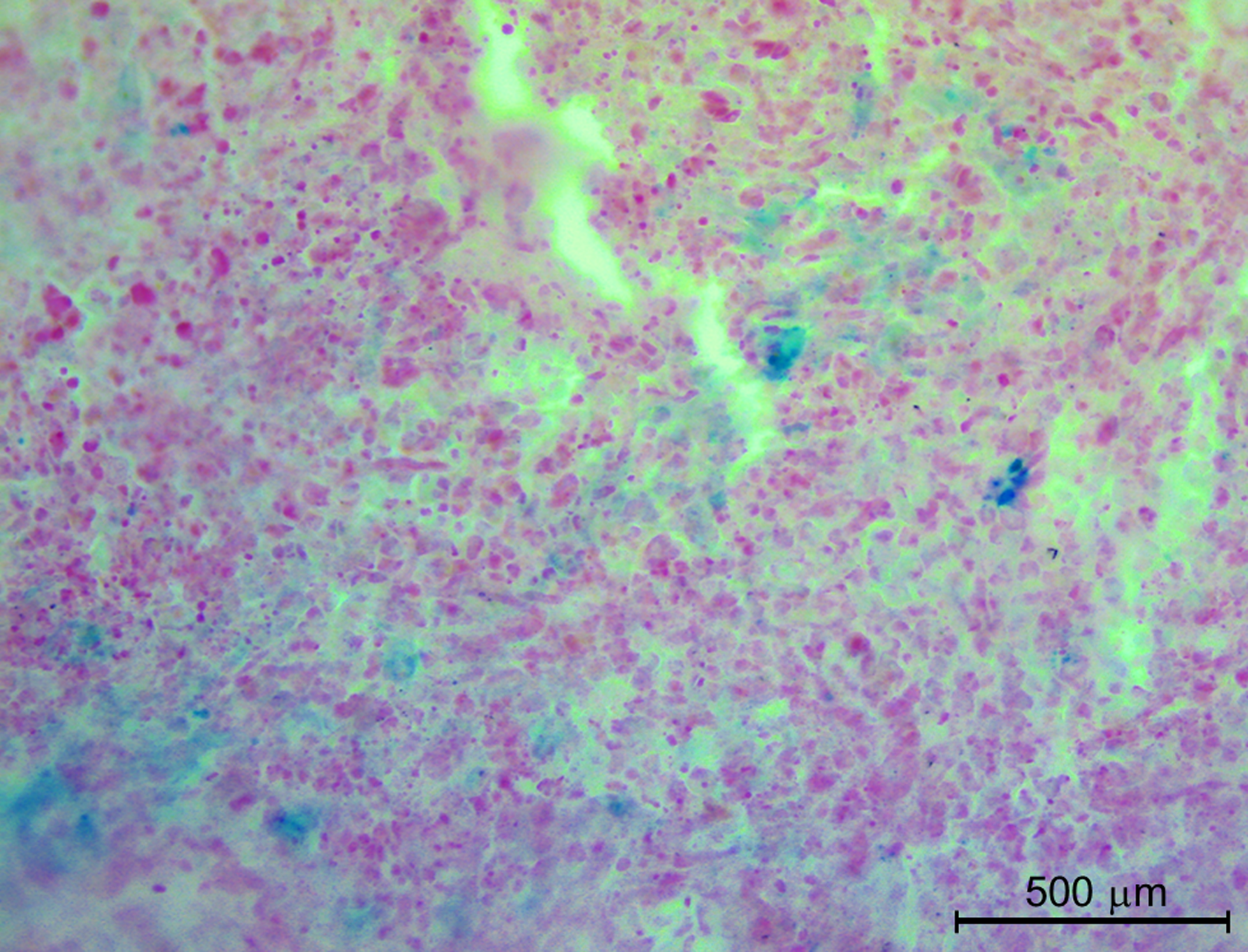

Supplement: Figure S5 — Prussian blue staining of a benign lymph node in an inflammation model, into which 10.4 mg Fe/kg of PJY10 was administered. Iron oxide particles within functional macrophages are stained blue (magnification×400). (TIF) [file pone.0107583.s005.tif]
