# Supplementary material for: Magnetic Resonance Imaging Diagnosis of Metastatic Lymph Nodes in a Rabbit Model: Efficacy of PJY10, a New Ultrasmall Superparamagnetic Iron Oxide Agent, with Monodisperse Iron Oxide Core and Multiple-Interaction Ligands
Source: PLoS One. 2014 Sep 12;9(9):e107583. doi: 10.1371/journal.pone.0107583 (PMC4162649; doi:10.1371/journal.pone.0107583)
Supplement: Table S1 — Pharmacokinetic Parameters of PJY10. (DOCX) [file pone.0107583.s006.docx]

**Table S1.** Pharmacokinetic parameters of PJY10.

| Group | Dose | Gender | K_el_ | T_1/2_ | AUC_inf_ | T_max_ | C_max_ | CL |
| --- | --- | --- | --- | --- | --- | --- | --- | --- |
|  | (mg Fe/kg) |  | (1/h) | (h) | (μg Fe⋅h/mL) | (h) | (μg Fe/mL) | (mL/h/kg) |
| T1 | 5.2 | Male | 0.34 | 2.0 | 33.9 | 0.033 | 39.9 | 153.6 |
| T2 | 10.4 | Male | 0.33 | 2.1 | 89.7 | 0.033 | 73.0 | 115.9 |
| T3 | 20.8 | Male | 0.34 | 2.1 | 290.9 | 0.033 | 158.2 | 71.5 |

Data are derived from plasma concentration-time curves. K_el_ = apparent terminal elimination rate constant, T_1/2_ = terminal half-life, AUC_inf_ = area under the plasma concentration-time curve from 0 h to infinity, with an extrapolation to the time infinity, T_max_ = time at which C_max_ is observed, C_max_ = maximum observed peak serum concentration, CL = total clearance.
