# Supplementary material for: Magnetic Resonance Imaging Diagnosis of Metastatic Lymph Nodes in a Rabbit Model: Efficacy of PJY10, a New Ultrasmall Superparamagnetic Iron Oxide Agent, with Monodisperse Iron Oxide Core and Multiple-Interaction Ligands
Source: PLoS One. 2014 Sep 12;9(9):e107583. doi: 10.1371/journal.pone.0107583 (PMC4162649; doi:10.1371/journal.pone.0107583)
Supplement: Text S1 — Pharmacokinetic Properties of PJY10. Sensitivity and Specificity for Detection of Lymph Node Metastasis: Comparison of PJY10 with MION-47. (DOCX) [file pone.0107583.s007.docx]

**Pharmacokinetic Properties of PJY10**

1) Blood half-life:

– 36 SPF Sprague-Dawley (SD) male rats weighing 260-340g were divided into three groups (ie, T1-3; 12 each) and PJY10 was intravenously administered via their tail veins as follows: 5.2 mg Fe/kg to T1; 10.4 mg Fe/kg to T2; and 20.8 mg Fe/kg to T3. The concentrations of PJY10 were derived from the T2 relaxation times of the plasma samples, which were measured at a 4.7T MR imager (Biospec 47/40; Bruker Medical Systems, Ettlingen, Germany). Additionally, pharmacokinetic parameters were derived from plasma concentration-time curves using WinNonlinTM (Ver. 5.2.1, Pharsight, Co., USA) software. Results are shown in Table S1 and Figure S2. Terminal half-life (T_1/2_) was 2.0-2.1 h in all three concentrations. Plasma concentration decreased linearly to less than 0.5 μg Fe/mL, limit of detection, 12-24 hours after the administration. No lethal adverse reaction occurred during the experiment.

2) Biodistribution:

– Biodistribution of PJY10 was studied using 36 6-week-old, male ICR mice. Prior to the administration, PJY10 nanoparticles were tagged with ^59^Fe by adding a small amount of ^59^FeCl_3_ in the first step of PJY10 synthesis. The ^59^Fe-tagged PJY10 nanoparticles (10.4 mg Fe/kg) were intravenously administered via their tail veins. At 2/5/10/30 minutes, 1/6 hours, 1/3/10/30/91/182 days following the administration, blood sampling and organ harvesting (muscle, bone, fat, heart, liver, spleen, lungs, kidneys, intestines, pancreas, stomach, inguinal/axillary/brachial lymph nodes) were performed. Three mice were sacrificed at each time point. Ratio of the residual ^59^Fe to the administered ^59^Fe (ie, %ID) was measured with a gamma counter and subsequently %ID per gram of the organ (%ID/g) as well as %ID per organ as a whole (%ID/organ) were calculated for each organ. Changes in %ID/organ and %ID/g with respect to time are demonstrated in Figure S3. Major ^59^Fe uptakes (%ID/g) were observed in the lymph nodes, blood, liver, and spleen. Specifically, %ID/g of the lymph nodes showed an overall tendency to increase in the first three to ten days and decrease thereafter.

**Sensitivity and Specificity for Detection of Lymph Node Metastasis: Comparison of PJY10 with MION-47**

Sensitivity and specificity for detection of lymph node metastasis were compared between the two groups (PJY10 group [10.4 mg Fe/kg] vs. MION-47 group [2.6 mg Fe/kg]) using Fischer exact test. Sensitivity of the PJY10 group was higher than that of the MION-47 group; however, it did not reach the statistical significance (100% [18 of 18] vs. 77% [10 of 13], *P* = .064). There was no significant difference in the specificity of the two groups (89% [47 of 53] vs. 75% [18 of 24], *P* = .175). Figure S4 represents a true-positive case of the metastatic lymph nodes.

For more accurate comparison, however, it would be more appropriate to use paired data by assigning the same individuals to both groups. Nonetheless, the two different USPIO particles could not be administered to the same individual rabbits considering their relatively long clearance times.
